# 2-Chloro-*N*-(3-chloro­phen­yl)acetamide

**DOI:** 10.1107/S1600536809011660

**Published:** 2009-04-02

**Authors:** B. Thimme Gowda, Sabine Foro, Hiromitsu Terao, Hartmut Fuess

**Affiliations:** a Department of Chemistry, Mangalore University, Mangalagangotri 574 199, Mangalore, India; b Institute of Materials Science, Darmstadt University of Technology, Petersenstrasse 23, D-64287 Darmstadt, Germany; c Faculty of Integrated Arts and Sciences, Tokushima University, Minamijosanjima-cho, Tokushima 770-8502, Japan

## Abstract

The N—H bond in the title compound, C_8_H_7_Cl_2_NO, is *anti* to the *meta*-chloro substituent in the aromatic ring in both independent mol­ecules comprising the asymmetric unit. The C=O bond is *anti* to the N—H bond and is also *anti* to the methyl­ene H atoms. Inter­molecular N—H⋯O hydrogen bonds link the mol­ecules into supra­molecular chains.

## Related literature

For preparation and characterisation of the compound, see: Pies *et al.* (1971 ▶), Gowda *et al.* (2006 ▶). For related structures, see: Gowda *et al.* (2008*a* ▶,*b* ▶,*c* ▶).
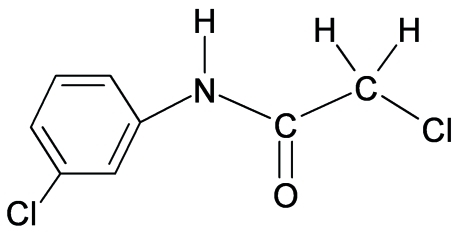


## Experimental

### 

#### Crystal data

C_8_H_7_Cl_2_NO
*M* *_r_* = 204.05
Orthorhombic, 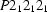

*a* = 4.897 (1) Å
*b* = 17.379 (3) Å
*c* = 21.484 (4) Å
*V* = 1828.4 (6) Å^3^
*Z* = 8
Mo *K*α radiation
μ = 0.66 mm^−1^
*T* = 299 K
0.45 × 0.08 × 0.02 mm

#### Data collection

Oxford Diffraction Xcalibur diffractometer with a Sapphire CCD detector
Absorption correction: multi-scan (*CrysAlis RED*; Oxford Diffraction, 2007 ▶) *T*_min_ = 0.756, *T*_max_ = 0.987
10213 measured reflections
3179 independent reflections
1745 reflections with *I* > 2σ(*I*)
*R*_int_ = 0.074

#### Refinement

*R*[*F* ^2^ > 2σ(*F* ^2^)] = 0.094
*wR*(*F* ^2^) = 0.103
*S* = 1.23
3179 reflections
217 parameters
H-atom parameters constrained
Δρ_max_ = 0.36 e Å^−3^
Δρ_min_ = −0.23 e Å^−3^
Absolute structure: Flack (1983 ▶), 1206 Friedel pairs
Flack parameter: 0.04 (13)

### 

Data collection: *CrysAlis CCD* (Oxford Diffraction, 2004 ▶); cell refinement: *CrysAlis RED* (Oxford Diffraction, 2007 ▶); data reduction: *CrysAlis RED*; program(s) used to solve structure: *SHELXS97* (Sheldrick, 2008 ▶); program(s) used to refine structure: *SHELXL97* (Sheldrick, 2008 ▶); molecular graphics: *PLATON* (Spek, 2009 ▶); software used to prepare material for publication: *SHELXL97*.

## Supplementary Material

Crystal structure: contains datablocks I, global. DOI: 10.1107/S1600536809011660/tk2407sup1.cif

Structure factors: contains datablocks I. DOI: 10.1107/S1600536809011660/tk2407Isup2.hkl

Additional supplementary materials:  crystallographic information; 3D view; checkCIF report

## Figures and Tables

**Table 1:** Hydrogen-bond geometry (Å, °)

*D*—H⋯*A*	*D*—H	H⋯*A*	*D*⋯*A*	*D*—H⋯*A*
N1—H1*N*⋯O2^i^	0.86	2.15	2.962 (6)	157
N2—H2*N*⋯O1^ii^	0.86	2.04	2.892 (6)	174

Symmetry codes: (i) 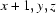; (ii) 
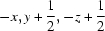
.

**Fig. 1.**
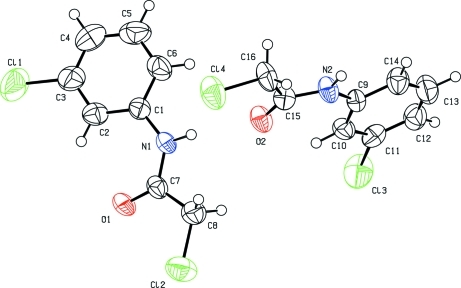
Molecular structure of (I), showing the atom labelling scheme. The displacement ellipsoids are drawn at the 50% probability level.

**Fig. 2.**
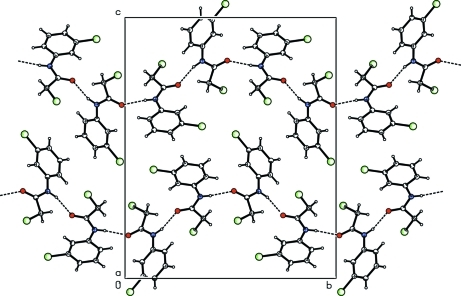
Molecular packing of (I) viewed in projection down the a-axis, with N-H···O hydrogen bonding shown as dashed lines.

## supplementary crystallographic information

### Comment

As part of a study of the effect of ring and side-chain substitutions on the solid-state geometry of aromatic amides (Gowda *et al.*, 2008*a, b, c*), in the present work the structure of 2-chloro-*N*-(3-chlorophenyl)acetamide (I) has been determined. The N—H bond is *anti* to the *meta*-chloro substituent in the aromatic ring (Fig. 1), similar to that observed in *N*-(3-chlorophenyl)acetamide (Gowda *et al.*, 2008*a*), but in contrast to the *syn* conformations observed with respect to the *meta*-methyl group in 2-chloro-*N*-(3-methylphenyl)acetamide (Gowda *et al.*, 2008*c*) and with respect to both chloro substituents in 2-chloro-*N*-(2,3-dichlorophenyl)acetamide (Gowda *et al.*, 2008*b*). Further, the C=O bond is not only *anti* to the N—H bond but also to the methylene-H-atoms. The asymmetric unit of the structure contains two molecules that are orthogonal to each other. The molecules in (I) are linked into infinite chains through intermolecular N1—H1···O2 and N2—H2—O1 hydrogen bonding (Table 1) as viewed down the a-axis (Fig. 2).

### Experimental

Compound (I) was prepared according to the literature method (Gowda *et al.*, 2006). The purity of (I) was checked by determining its melting point and characterised by recording its infrared, NMR and NQR spectra (Gowda *et al.*, 2006 & Pies *et al.*, 1971). Single crystals of (I) were obtained from an ethanolic solution held at room temperature.

### Refinement

The H atoms were positioned with idealised geometry using a riding model with C—H = 0.93–0.97 Å and N—H = 0.86 Å, and were refined with isotropic displacement parameters set to 1.2 x *U*_eq_(C).

### Crystal data

**Table:**

C_8_H_7_Cl_2_NO	*F*(000) = 832
*M**_r_* = 204.05	*D*_x_ = 1.483 Mg m^−^^3^
Orthorhombic, *P*2_1_2_1_2_1_	Mo *K*α radiation, λ = 0.71073 Å
Hall symbol: P 2ac 2ab	Cell parameters from 2170 reflections
*a* = 4.897 (1) Å	θ = 2.2–27.3°
*b* = 17.379 (3) Å	µ = 0.66 mm^−^^1^
*c* = 21.484 (4) Å	*T* = 299 K
*V* = 1828.4 (6) Å^3^	Needle, colourless
*Z* = 8	0.45 × 0.08 × 0.02 mm

### Data collection

**Table:**

Oxford Diffraction Xcalibur diffractometer with a Sapphire CCD detector	3179 independent reflections
Radiation source: fine-focus sealed tube	1745 reflections with *I* > 2σ(*I*)
graphite	*R*_int_ = 0.074
Rotation method data acquisition using ω and φ scans	θ_max_ = 25.3°, θ_min_ = 2.2°
Absorption correction: multi-scan (*CrysAlis RED*; Oxford Diffraction, 2007)	*h* = −5→5
*T*_min_ = 0.756, *T*_max_ = 0.987	*k* = −20→20
10213 measured reflections	*l* = −25→23

### Refinement

**Table:**

Refinement on *F*^2^	Secondary atom site location: difference Fourier map
Least-squares matrix: full	Hydrogen site location: inferred from neighbouring sites
*R*[*F*^2^ > 2σ(*F*^2^)] = 0.094	H-atom parameters constrained
*wR*(*F*^2^) = 0.103	*w* = 1/[σ^2^(*F*_o_^2^) + (0.0084*P*)^2^ + 1.6742*P*] where *P* = (*F*_o_^2^ + 2*F*_c_^2^)/3
*S* = 1.23	(Δ/σ)_max_ = 0.005
3179 reflections	Δρ_max_ = 0.36 e Å^−^^3^
217 parameters	Δρ_min_ = −0.23 e Å^−^^3^
0 restraints	Absolute structure: Flack (1983), 1206 Friedel pairs
Primary atom site location: structure-invariant direct methods	Flack parameter: 0.04 (13)

### Special details

**Table:**

Geometry. All e.s.d.'s (except the e.s.d. in the dihedral angle between two l.s. planes) are estimated using the full covariance matrix. The cell e.s.d.'s are taken into account individually in the estimation of e.s.d.'s in distances, angles and torsion angles; correlations between e.s.d.'s in cell parameters are only used when they are defined by crystal symmetry. An approximate (isotropic) treatment of cell e.s.d.'s is used for estimating e.s.d.'s involving l.s. planes.
Refinement. Refinement of *F*^2^ against ALL reflections. The weighted *R*-factor *wR* and goodness of fit *S* are based on *F*^2^, conventional *R*-factors *R* are based on *F*, with *F* set to zero for negative *F*^2^. The threshold expression of *F*^2^ > σ(*F*^2^) is used only for calculating *R*-factors(gt) *etc*. and is not relevant to the choice of reflections for refinement. *R*-factors based on *F*^2^ are statistically about twice as large as those based on *F*, and *R*- factors based on ALL data will be even larger.

### Fractional atomic coordinates and isotropic or equivalent isotropic displacement parameters (Å^2^)

**Table:**

	*x*	*y*	*z*	*U*_iso_*/*U*_eq_	
Cl1	0.6906 (6)	0.02452 (11)	−0.05228 (9)	0.1205 (10)	
Cl2	−0.0094 (4)	0.03432 (10)	0.27874 (9)	0.0775 (6)	
O1	0.3155 (10)	0.0159 (2)	0.16486 (19)	0.0612 (14)	
N1	0.5179 (11)	0.1333 (3)	0.1663 (2)	0.0466 (14)	
H1N	0.5335	0.1741	0.1887	0.056*	
C1	0.6654 (15)	0.1331 (3)	0.1102 (3)	0.0429 (17)	
C2	0.6148 (14)	0.0817 (3)	0.0628 (3)	0.052 (2)	
H2	0.4813	0.0441	0.0673	0.063*	
C3	0.7641 (19)	0.0868 (4)	0.0088 (3)	0.065 (2)	
C4	0.9656 (17)	0.1404 (5)	0.0005 (4)	0.071 (2)	
H4	1.0676	0.1422	−0.0360	0.086*	
C5	1.0113 (16)	0.1917 (5)	0.0482 (4)	0.076 (2)	
H5	1.1442	0.2295	0.0436	0.091*	
C6	0.8643 (15)	0.1880 (4)	0.1025 (3)	0.059 (2)	
H6	0.8996	0.2229	0.1343	0.071*	
C7	0.3544 (15)	0.0775 (4)	0.1898 (3)	0.0451 (18)	
C8	0.2235 (15)	0.1014 (3)	0.2507 (3)	0.062 (2)	
H8A	0.1310	0.1502	0.2449	0.074*	
H8B	0.3655	0.1089	0.2816	0.074*	
Cl3	0.3476 (5)	0.13531 (10)	0.41715 (9)	0.0882 (7)	
Cl4	−0.7058 (4)	0.32439 (10)	0.17867 (9)	0.0819 (7)	
O2	−0.2983 (9)	0.2490 (2)	0.25851 (18)	0.0523 (12)	
N2	−0.1818 (11)	0.3561 (2)	0.3135 (2)	0.0484 (14)	
H2N	−0.2216	0.4041	0.3169	0.058*	
C9	0.0161 (13)	0.3283 (4)	0.3558 (3)	0.0400 (16)	
C10	0.0721 (13)	0.2514 (4)	0.3645 (3)	0.0468 (18)	
H10	−0.0243	0.2138	0.3430	0.056*	
C11	0.2759 (15)	0.2317 (4)	0.4062 (3)	0.0484 (19)	
C12	0.4158 (14)	0.2849 (4)	0.4398 (3)	0.058 (2)	
H12	0.5517	0.2702	0.4676	0.069*	
C13	0.3504 (17)	0.3615 (4)	0.4315 (3)	0.069 (2)	
H13	0.4408	0.3989	0.4547	0.083*	
C14	0.1548 (16)	0.3833 (4)	0.3897 (3)	0.057 (2)	
H14	0.1150	0.4351	0.3841	0.068*	
C15	−0.3165 (14)	0.3176 (4)	0.2684 (3)	0.0442 (16)	
C16	−0.4892 (14)	0.3714 (3)	0.2297 (3)	0.0599 (19)	
H16A	−0.3697	0.4050	0.2061	0.072*	
H16B	−0.5973	0.4033	0.2573	0.072*	

### Atomic displacement parameters (Å^2^)

**Table:**

	*U*^11^	*U*^22^	*U*^33^	*U*^12^	*U*^13^	*U*^23^
Cl1	0.221 (3)	0.0728 (13)	0.0674 (14)	−0.0099 (18)	0.0240 (19)	−0.0174 (12)
Cl2	0.0703 (15)	0.0686 (12)	0.0935 (15)	−0.0124 (13)	0.0192 (14)	0.0007 (12)
O1	0.089 (4)	0.030 (2)	0.064 (3)	−0.017 (3)	0.007 (3)	−0.011 (2)
N1	0.060 (4)	0.031 (3)	0.049 (4)	−0.009 (3)	0.001 (3)	−0.008 (3)
C1	0.051 (5)	0.031 (4)	0.046 (4)	0.005 (4)	−0.001 (4)	0.000 (4)
C2	0.058 (6)	0.041 (4)	0.058 (5)	0.005 (4)	0.005 (4)	0.006 (4)
C3	0.090 (7)	0.044 (4)	0.061 (5)	0.005 (5)	0.003 (5)	0.008 (4)
C4	0.071 (6)	0.083 (6)	0.061 (6)	0.019 (6)	0.012 (5)	0.013 (5)
C5	0.055 (6)	0.094 (7)	0.080 (6)	−0.016 (5)	0.007 (6)	0.022 (6)
C6	0.047 (5)	0.063 (5)	0.069 (6)	−0.011 (5)	−0.015 (5)	0.004 (4)
C7	0.050 (5)	0.034 (4)	0.051 (4)	−0.001 (4)	−0.012 (4)	0.000 (4)
C8	0.079 (6)	0.047 (4)	0.059 (5)	−0.008 (4)	0.013 (5)	−0.007 (3)
Cl3	0.125 (2)	0.0597 (12)	0.0799 (14)	0.0279 (14)	−0.0236 (14)	0.0066 (11)
Cl4	0.0896 (16)	0.0693 (12)	0.0869 (14)	0.0000 (13)	−0.0394 (13)	−0.0053 (11)
O2	0.061 (3)	0.032 (2)	0.064 (3)	0.006 (3)	−0.012 (3)	−0.009 (2)
N2	0.056 (4)	0.031 (3)	0.058 (4)	0.007 (3)	−0.009 (3)	−0.013 (3)
C9	0.037 (4)	0.043 (4)	0.040 (4)	0.000 (4)	−0.004 (4)	−0.006 (4)
C10	0.053 (5)	0.035 (4)	0.052 (4)	−0.003 (4)	0.002 (4)	−0.001 (3)
C11	0.055 (5)	0.050 (4)	0.040 (4)	0.012 (4)	0.004 (4)	0.005 (3)
C12	0.049 (6)	0.073 (5)	0.051 (5)	0.000 (4)	−0.007 (4)	−0.003 (4)
C13	0.079 (6)	0.061 (5)	0.066 (6)	−0.007 (5)	−0.011 (5)	−0.016 (4)
C14	0.067 (6)	0.049 (4)	0.055 (5)	0.007 (5)	−0.009 (4)	−0.006 (4)
C15	0.046 (4)	0.040 (4)	0.047 (4)	0.010 (4)	−0.004 (4)	−0.001 (4)
C16	0.059 (5)	0.050 (4)	0.071 (5)	−0.001 (4)	−0.029 (5)	−0.011 (4)

### Geometric parameters (Å, °)

**Table:**

Cl1—C3	1.739 (7)	Cl3—C11	1.728 (6)
Cl2—C8	1.738 (6)	Cl4—C16	1.730 (6)
O1—C7	1.212 (6)	O2—C15	1.215 (6)
N1—C7	1.354 (7)	N2—C15	1.349 (7)
N1—C1	1.406 (7)	N2—C9	1.415 (7)
N1—H1N	0.8600	N2—H2N	0.8600
C1—C6	1.373 (8)	C9—C10	1.376 (7)
C1—C2	1.376 (7)	C9—C14	1.380 (8)
C2—C3	1.375 (8)	C10—C11	1.384 (8)
C2—H2	0.9300	C10—H10	0.9300
C3—C4	1.369 (9)	C11—C12	1.359 (8)
C4—C5	1.376 (9)	C12—C13	1.381 (8)
C4—H4	0.9300	C12—H12	0.9300
C5—C6	1.373 (9)	C13—C14	1.366 (8)
C5—H5	0.9300	C13—H13	0.9300
C6—H6	0.9300	C14—H14	0.9300
C7—C8	1.516 (8)	C15—C16	1.510 (8)
C8—H8A	0.9700	C16—H16A	0.9700
C8—H8B	0.9700	C16—H16B	0.9700
			
C7—N1—C1	128.4 (5)	C15—N2—C9	128.9 (5)
C7—N1—H1N	115.8	C15—N2—H2N	115.6
C1—N1—H1N	115.8	C9—N2—H2N	115.6
C6—C1—C2	119.3 (6)	C10—C9—C14	120.2 (6)
C6—C1—N1	117.8 (6)	C10—C9—N2	123.7 (6)
C2—C1—N1	122.9 (7)	C14—C9—N2	116.1 (6)
C1—C2—C3	119.1 (7)	C9—C10—C11	118.1 (6)
C1—C2—H2	120.5	C9—C10—H10	120.9
C3—C2—H2	120.5	C11—C10—H10	120.9
C4—C3—C2	122.5 (7)	C12—C11—C10	122.6 (6)
C4—C3—Cl1	118.3 (7)	C12—C11—Cl3	119.0 (6)
C2—C3—Cl1	119.1 (7)	C10—C11—Cl3	118.3 (6)
C3—C4—C5	117.4 (8)	C11—C12—C13	118.1 (7)
C3—C4—H4	121.3	C11—C12—H12	121.0
C5—C4—H4	121.3	C13—C12—H12	121.0
C6—C5—C4	121.2 (8)	C14—C13—C12	120.9 (7)
C6—C5—H5	119.4	C14—C13—H13	119.5
C4—C5—H5	119.4	C12—C13—H13	119.5
C5—C6—C1	120.4 (7)	C13—C14—C9	120.0 (7)
C5—C6—H6	119.8	C13—C14—H14	120.0
C1—C6—H6	119.8	C9—C14—H14	120.0
O1—C7—N1	124.1 (6)	O2—C15—N2	125.2 (6)
O1—C7—C8	123.8 (6)	O2—C15—C16	123.5 (6)
N1—C7—C8	112.1 (5)	N2—C15—C16	111.3 (5)
C7—C8—Cl2	113.2 (4)	C15—C16—Cl4	113.6 (4)
C7—C8—H8A	108.9	C15—C16—H16A	108.8
Cl2—C8—H8A	108.9	Cl4—C16—H16A	108.8
C7—C8—H8B	108.9	C15—C16—H16B	108.8
Cl2—C8—H8B	108.9	Cl4—C16—H16B	108.8
H8A—C8—H8B	107.8	H16A—C16—H16B	107.7
			
C7—N1—C1—C6	−165.6 (6)	C15—N2—C9—C10	−11.4 (10)
C7—N1—C1—C2	15.7 (10)	C15—N2—C9—C14	169.1 (6)
C6—C1—C2—C3	−0.3 (9)	C14—C9—C10—C11	−2.2 (9)
N1—C1—C2—C3	178.4 (6)	N2—C9—C10—C11	178.3 (5)
C1—C2—C3—C4	1.1 (10)	C9—C10—C11—C12	1.9 (9)
C1—C2—C3—Cl1	−177.3 (5)	C9—C10—C11—Cl3	−179.8 (5)
C2—C3—C4—C5	−1.6 (11)	C10—C11—C12—C13	−0.1 (10)
Cl1—C3—C4—C5	176.8 (6)	Cl3—C11—C12—C13	−178.4 (5)
C3—C4—C5—C6	1.3 (11)	C11—C12—C13—C14	−1.4 (11)
C4—C5—C6—C1	−0.6 (11)	C12—C13—C14—C9	1.1 (11)
C2—C1—C6—C5	0.0 (10)	C10—C9—C14—C13	0.7 (10)
N1—C1—C6—C5	−178.7 (6)	N2—C9—C14—C13	−179.7 (6)
C1—N1—C7—O1	1.8 (10)	C9—N2—C15—O2	3.9 (11)
C1—N1—C7—C8	−177.8 (6)	C9—N2—C15—C16	−174.2 (5)
O1—C7—C8—Cl2	−4.6 (9)	O2—C15—C16—Cl4	10.9 (9)
N1—C7—C8—Cl2	175.0 (4)	N2—C15—C16—Cl4	−170.9 (4)

### Hydrogen-bond geometry (Å, °)

**Table:**

*D*—H···*A*	*D*—H	H···*A*	*D*···*A*	*D*—H···*A*
N1—H1N···O2^i^	0.86	2.15	2.962 (6)	157
N2—H2N···O1^ii^	0.86	2.04	2.892 (6)	174

Symmetry codes: (i) *x*+1, *y*, *z*; (ii) −*x*, *y*+1/2, −*z*+1/2.
